# Symmetry Criterion for Patients with Rheumatoid Arthritis of the Foot: A Cross-Sectional Study

**DOI:** 10.3390/ijerph18073619

**Published:** 2021-03-31

**Authors:** Jose Alberto Sanchez-Castillo, Andres Reinoso-Cobo, Gabriel Gijon-Nogueron, Rafael Caliz-Caliz, Manuela Exposito-Ruiz, Laura Ramos-Petersen, Ana Belen Ortega-Avila

**Affiliations:** 1 Department of Nursing and Podiatry, Faculty of Health Sciences, University of Malaga, 29071 Málaga, Spain; jasanchezc@uma.es (J.A.S.-C.); andreicob@uma.es (A.R.-C.); anaortavi@uma.es (A.B.O.-A.); 2 Biomedical Research Institute (IBIMA), 29010 Malaga, Spain; 3 Department of Rheumatology, Virgen de las Nieves University Hospital, 18014 Granada, Spain; antonior.caliz.sspa@juntadeandalucia.es; 4 Departamento de Estadística e Investigación Operativa, Universidad de Granada, 18100 Granada, Spain; mexpositoruiz@ugr.es; 5 Department of Podiatry, Faculty of Health Sciences, Universidad Catolica San Antonio de Murcia, 30107 Murcia, Spain; lrpetersen@ucam.edu

**Keywords:** rheumatoid arthritis, foot health, symmetry, functionality, pain

## Abstract

Objective: The aim of the study was to analyze the feet of rheumatoid arthritis (RA) patients, to determine the degree to which both feet were affected, primarily analyzing the severity of RA in both feet looking at structure and morphology, and secondly looking at the symmetry in terms of the anthropometrics and posture. Method: This cross-sectional study was conducted from January to December 2018. The data from 229 patients with RA and with foot pain and no RA recruited (Granada, Spain) were analyzed. Two researchers independently interviewed the patients to obtain the study data. The clinical data were obtained using specific foot health and quality of life questionnaires and a validated platform for foot measurement. Anthropometric measurements were obtained by means of a foot measurement platform and the Foot Posture Index (FPI). The bivariate analysis was performed with the Student’s *t* test and the non-parametric Wilcoxon test. The level of significance was established at *p* < 0.05. Results: In the RA group, anthropometric measurements revealed significant differences between the left and right feet in 13 of the 23 parameters considered, as follows: (non-load-bearing) foot length, length of the first metatarsophalangeal joint, maximum height of the internal longitudinal arch, and width of the midfoot (*p* < 0.001, *p* = 0.038, *p* < 0.001, and *p* = 0.037 respectively); and Foot Posture Index (*p* = 0.001). Conclusions: In patients with RA, statistically significant differences were found in the Foot Posture Index and in several parameters related to foot structure and morphology. From this, we conclude that from a morphological, structural, and postural standpoint, a pattern of symmetric joint involvement should not be viewed as a specific criterion for RA in the foot.

## 1. Introduction

Rheumatoid arthritis (RA) is a common form of inflammatory arthritis that is most prevalent in North America and in Europe [1], where it affects 0.5–1% of the population [2]. The most characteristic symptom is inflammation, which provokes significant changes in joint structures, limits their function [3], and is associated with increased mortality and morbidity [1].

RA mainly affects the small joints of the hands and feet, and 50% of patients experience foot problems within three years of diagnosis [4]. The most frequent deformations in the foot affect the forefoot and the hindfoot, with pathologies such as hallux valgus, metatarsal subluxation, and hammer or claw toes [5]. Signs of RA often appear at an early stage in the forefoot, and problems there develop quickly [6]. RA-related pathologies appear as the joints and ligaments deteriorate [7], limiting movement in the ankle and foot. This development produces an unequal distribution of pressures and makes it painful to remain in a standing position [8].

According to most authors, the joint damage provoked by RA is largely symmetric, and the first symptoms are considered to be symmetric polyarthritis of the small joints, together with pain, inflammation, and stiffness of the hand and/or foot. However, although symmetry is often said to be a prominent feature of RA [4,9,10,11,12], there is no scientific evidence to support this view. In this respect, Zangger et al. reported a general prevalence of asymmetry in joint damage in RA (of approximately 13–16%) and a tendency for this asymmetry to increase slightly as the disease progresses [13]. Furthermore, Aletaha et al., in their review of classification criteria for RA, in 2010, rejected symmetry as a specific diagnostic criterion [14].

To our knowledge, the present study is the first to analyze the symmetric characteristics of the foot in patients with RA, from an anthropometric standpoint and taking foot posture into account. We hypothesize that, following the symmetrical pattern of joint involvement in RA, structural and morphological alterations in terms of both feet anthropometrics and posture should be symmetrical as well. The aim of the study was to analyze the feet of rheumatoid arthritis (RA) patients, to determine the degree to which both feet were affected, primarily analzsing the severity of RA in both feet looking at structure and morphology, and secondly looking at the symmetry in terms of the anthropometrics and posture.

## 2. Materials and Methods

### 2.1. Ethical Approval

This study was performed in accordance with the Declaration of Helsinki and was approved by the ethics committees of the University of Malaga and the Junta de Andalucía (CEUMA-91-2015-H y PEIBA-ARC0001).

### 2.2. Study Design

The Strengthening the Reporting of Observational Studies in Epidemiology (STROBE) criteria were followed in this secondary analysis of a cross-sectional study.

### 2.3. Participants

A convenience sample was obtained from 229 patients with foot pain and RA according to the 2010 Rheumatoid Arthritis Classification Criteria (approved by the American College of Rheumatology and the European League Against Rheumatism) [14] and from 64 patients with foot pain but without RA. The patients were recruited at a hospital outpatient service for the RA group and at podiatry clinics for those with no RA, from January to December 2018. All these participants had a history of subtalar and/or ankle and/or talonavicular or hindfoot pain producing a pain level of over 3 on a Visual Analysis Scale (VAS) pain scale [15]. None made regular use of walking aids and all were able to perform at least the minimum range of movements in the ankle, subtalar, and mid-tarsal joints required to walk normally. The exclusion criteria applied were the presence of concomitant musculoskeletal disease, central or peripheral nervous system disease, or endocrine disorders (especially diabetes mellitus).

RA patients who met the inclusion criteria were contacted by members of the rheumatology service at the Virgen de las Nieves Hospital (Granada, Spain), given an information sheet, and invited to participate. Patients with foot pain but no RA were recruited at private podiatry clinics in Granada and Malaga and at the Podiatry Attention Unit of the University of Malaga. Those who agreed were interviewed and given further information about the study. All participants gave signed informed consent before being interviewed.

### 2.4. Procedure

Two researchers (A.R.C. and G.G.N.), working independently, interviewed the participants to obtain the necessary data. In all cases, demographic and health data (VAS, both general [16], and specific to the foot and hand), Disease Activity Score-28 (DAS28), and anthropometric measurements of the foot were collected. For the patients with RA, current treatment details, including the duration of the illness, were also recorded.

**Figure 1:**
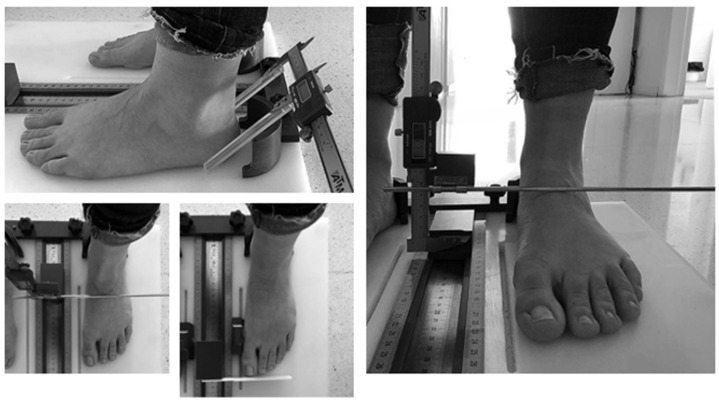
Anthropometric measurements of the foot using a validated measurement platform [17].

For the anthropometric measurements, a foot measurement platform, validated by McPoil et al. [17], was used to measure foot length, midfoot, forefoot, and hindfoot width and midfoot height (load-bearing and non-load-bearing) (Figure 1).

Each participant was asked to stand on the platform to obtain the load-bearing measurement and then to sit down for the non-load-bearing measurement. In both cases, the body weight was evenly distributed between the both feet, the patient was stood in a relaxed standing position, looking straight ahead, with weight evenly distributed between both feet. The measurements were taken with the patient’s feet in the heel cups of the measurement platform, with the heels as far back as possible, and with the first metatarsal heads against the surface boundary. One of the researchers (A.R.C.) conducted a repeatability and reliability analysis of this procedure, with 30 participants (intraclass correlation coefficient (ICC) for the instrument, 0.96–0.98). In addition, the anthropometric measurements was analyzed with the equation Zifchock et al. [18] (((measurement left-measurement right)/measurement left) *100) consistent with previous research, the left side was chosen for the Symmetry Index (SI) equation with a single side as a reference value as opposed to the right side, and foot length, midfoot width, forefoot width and maximum height of the internal longitudinal arch were included.

Foot posture was also determined using the Foot Posture Index (FPI), which is known to be a reliable instrument for this purpose (ICC for the clinician, 0.94–0.96) [19]. This measurement was evaluated by another researcher (G.G.N.), producing an ICC of 0.95–0.97.

The information thus obtained was recorded in a database by another of the researchers (A.S.C.), who was blinded to the group membership by means of a coding system only available to the principal investigator (A.B.O.A.).

### 2.5. Statistical Analysis

The results obtained are reported as the median and interquartile range, when the distribution of the variables was non-normal and as means and standard deviations (mean ± SD) when the distribution was normal. The Kolmogorov–Smirnov test was applied to determine the normality or otherwise of the distributions, and the reliability of the measuring instruments was calculated with an ICC model. The bivariate analysis was performed with Student’s *t* test and the non-parametric Wilcoxon test. The level of significance was established at *p* < 0.05. All statistical analyses were performed using SPSS v.24.08 statistical software (SPSS Inc., Chicago, IL, USA).

## 3. Results

In all, 316 patients were contacted, provided with information about the study and invited to participate. Of these, 17 refused to participate, leaving a study population of 299 persons, of whom 235 patients were included in the RA group and 64 in the control group. In the RA group, 173 of the patients (73.61%) were women, as were 50 (78.12%) in the control group. The mean age of the patients with RA was 52.97 years (SD: 13.60), and in the control group, it was 58.39 years (SD: 12.73). The mean values for height and weight were 163.91 cm (SD: 7.74) and 68.52 kg (SD: 12.12), respectively, and body mass index (BMI) was 25.47(SD:10.2) for the RA group, and 162.49 cm (SD: 11.01), 71.28 kg (SD: 14.65), and BMI 27.17(SD:12.57), respectively, for the control group.

In the RA group, 92 patients (39.1%) had had the disease for up to ten years, while 143 (60.9%) had had it for over ten years. The patients with RA were under different pharmacological management: biological disease-modifying antirheumatic drugs (bDMARDs) (42%), methotrexate (35%), or nonsteroidal anti-inflammatory drugs (NSAIDs)/corticosteroids (20%). The DAS28 was measured to control the disease activity, and the score was 3.15 (SD 1.4) within patients with less than 10 years of disease activity and 2.85 (SD 1.18) within patients with more than 10 years of disease activity.

Table 1 shows the detailed results obtained for anthropometric and foot posture measurements (without taking into account the duration of the disease), for each foot separately, both for the RA group and for the control group.

Among the patients with RA (without taking into account the duration of the disease), the anthropometric measurements revealed significant differences between the left and right feet in 13 of the 23 parameters analyzed, as follows: foot length, both load-bearing and non-load-bearing (*p* < 0.001); length of the first metatarsophalangeal joint, non-load-bearing and load-bearing (*p* = 0.038 and *p* = 0.011, respectively); maximum height of the internal longitudinal arch (ILA), both non-load-bearing and load-bearing (*p* < 0.001); difference in maximum height of the ILA (*p* = 0.001); midfoot width, non-load-bearing and load-bearing (*p* = 0.037 and *p* < 0.001, respectively); navicular drop, non-load-bearing and load-bearing (*p* = 0.010 and *p* = 0.002, respectively); and stiffness (*p* < 0.001).

Furthermore, the maximum height of the ILA in the right foot was significantly lower than in the left foot, both load-bearing and non-load-bearing (*p* = 0.001). In contrast, in the control group, there was no statistically significant difference in this respect (*p* = 0.446).

In addition, the statistician used the Symmetry Index to measure the maximum height of the internal longitudinal arch, which showed a statistically significant difference (*p* < 0.01). (Table 2)

A similar contrast was observed with respect to foot posture. Thus, the patients in the RA group presented statistically significant differences (*p* = 0.001) in the FPI between the right and left feet, with mean values of 5 and 6, respectively. No such difference was observed in the control group (*p* = 0.273).

The anthropometry measurements and the FPI results for the RA group, taking into account the duration of the disease, are shown in Table 3.

As can be seen in the table, when the duration of the RA condition was taken into account (≤10 years vs. >10 years), there were statistically significant differences between the left and right feet in the following respects: foot length, non-load-bearing (*p* = 0.005 vs. *p* = 0.002, respectively; and load-bearing (*p* < 0.001 vs. *p* = 0.005); maximum height of the ILA, non-load-bearing (*p* < 0.001 vs. *p* < 0.001) and load-bearing (*p* < 0.001 vs. *p* < 0.001); difference in the maximum height of the ILA (*p* = 0.017 vs. *p* = 0.021), but if the Symmetry Index was used (Table 4), there was no statistically significant difference between groups.

Regarding foot posture, the patients who had had RA for up to ten years presented significant differences (*p* = 0.002) between FPI values for the left and right feet. No such differences were observed among the patients who had been suffering from RA for more than ten years (*p* = 0.086).

## 4. Discussion

The aim of this study is to analyze and compare the degree of symmetry observed in the feet of patients with RA, in terms of anthropometry and posture. According to the results obtained, patients with RA, irrespective of the duration of the disease, presented statistically significant differences between the left and right feet in 13 of the 23 morphological, structural, and postural parameters analyzed, namely foot length, length of the first metatarsophalangeal joint, midfoot width and navicular drop (in every case, both load-bearing and non-load-bearing), stiffness, the FPI, the maximum height of the ILA (both load-bearing and non-load-bearing), and in the differences in maximum ILA height. The latter question is of particular interest as it is one of the causes of the development of flat feet in these patients. In the control group, these differences were observed in only 6 of the 23 parameters. As concerns the FPI, there were no statistically significant differences between the left and right feet. In other words, in the control group, the left and right feet were morphologically and structurally equivalent. Thus, although the symmetric involvement of the joints is often considered a characteristic feature of RA [4,9,10,11,12], this association is not confirmed by our study findings.

Our results are in line with those obtained by Helliwell et al. [20], who studied a group of patients with characteristics similar to ours (67% women, average age 55.2 years and 60 years in the groups with RA; *n* = 103). These authors concluded that the presence of symmetric involvement of the joints cannot be used to distinguish RA from other forms of inflammatory arthritis such as psoriatic arthritis. This view has been corroborated by Aletaha el al. [14], who reported that symmetry was not of significant importance according to an analysis of their study data. In this case, too, the population samples considered had similar characteristics to our own study groups (66.8% women, average age 51 years in the groups with RA, *n* = 3115).

On the other hand, a fact that seems significant (and one that might justify further investigation) is that in our RA group, the right foot presented significantly lower average values than the left for maximum ILA height, both load-bearing and non-load-bearing, and therefore, the difference was smaller in each case. These results are in line with those obtained by Kuryliszyn-Mopskal et al. [21], who found that flattening of the lateral arch in the right foot is statistically much more frequent in patients with RA than in those with osteoarthritis and in the control group. In addition, there were statistically significant differences in the values of the hallux valgus angle of the right foot in the patients with RA versus those with osteoarthritis and those in the control group. However, it should be noted that the sample in question was composed exclusively of women (*n* = 94).

Among the patients who had had RA for up to ten years, significant differences were observed between the FPI values for the left and right feet. However, no such difference was found in the RA patients who had had the disease for longer or in the control group. Similarly, in the patients who had had RA for over ten years, only 8 of the 23 parameters considered presented significant differences between the left and right feet. This result is in line with that obtained by Zangger et al., who suggested there may be a trend towards symmetrization in the damage to the less affected joint, depending on the duration of the RA [13].

In contrast to the above-mentioned differences, with respect to foot posture we found no significant differences between the values for the left and right feet, either in the RA group or in the control group.

The present study is subject to certain limitations. Firstly, the population groups analyzed were heterogeneous, since the number of persons in the group with RA was significantly higher than in the control group (persons with foot pain but no RA), which may have influenced the findings obtained. Furthermore, no account was taken of leg dominance, and this too may have influenced the results. In addition, and as Bukhari et al. have pointed out, the clinical concept of symmetry in itself is difficult to define precisely, as it involves concepts such as what is understood by impact on the joints, and deciding which groups of joints should be taken into account [12]. Finally, it might be relevant that the majority of our study population were female, although this prevalence reflects that of the general population affected by RA and foot pain [22,23] In addition, the clinical manifestations of patients that develop symmetrical damage differs from the patients that developed asymmetrical damage but they were not obtained.

On the other hand, our study also presents important strengths. To our knowledge, this is the first cross-sectional analysis to be conducted of anthropometric and postural parameters of the foot, in relation to the proposed characteristic of symmetrical presentation of alterations in the joints of patients with RA. Nevertheless, further longitudinal studies are needed in order to take into account parameters such as the duration of the disease and the type of treatment received, both of which could influence the way in which different joints are affected. In further studies, analyzing more homogeneous sample sizes is needed, as a non-homogeneous sample size may influence the results. Furthermore, studies with outcomes that allow making a relationship between pain, loss of functionality and/or quality of life, and feet deformity are required. Finally, in order to achieve greater uniformity of criteria, a precise definition is needed of the concept of symmetry.

As commented above, the use of a symmetric pattern of involvement of the joints as a diagnostic criterion for RA could result in misdiagnosis in cases when such a pattern is absent. In our view, consideration of the study results presented may be useful in the clinical diagnosis of patients with RA of the foot, although more research is needed in order to improve the diagnostic criteria applied when RA is suspected.

## 5. Conclusions

The study results obtained show that, although a pattern of symmetric joint involvement is generally considered a characteristic clinical sign of RA, this assumption might lead to an incorrect diagnosis. In short, morphological, structural, and postural symmetry, as a clinical sign of RA in the joints, should not be taken as a specific criterion for patients with RA in the feet. In terms of anthropometry and posture, patients with RA, irrespective of the duration of the disease, presented statistically significant differences between the left and right feet, in the control group, the left and right feet were morphologically and structurally equivalent.

## Figures and Tables

**Table 1:** Left/Right foot comparison of patients with rheumatoid arthritis (RA) and of those with foot pain and no RA.

	RA Group			Non-RA Group		
	Right Foot	Left Foot		Right Foot	Left Foot	
	Mean/SD or Median/Interquartile Range *n* = 235		*p* Value	Mean/SD or Median/Interquartile Range *n* = 64		*p* Value
Foot length NLB (mm)	240 (231–251)	240 (232–251)	<0.001	241.81 ± 13.76	241.91 ± 13.18	0.749
First MTJ NLB (mm)	179 (171–186)	179 (172–187)	0.038	177.203 ± 10.27	176.94 ± 9.76	0.436
Max ILA NLB (mm)	57.66 (53.06–62.16)	59.89 (55.88–65.25)	<0.001	62 ± 9.11	63.3 ± 8.2	0.002
Midfoot width NLB (mm)	73.18 ± 6.41	73.66 ± 6.66	0.037	70.93 ± 6.35	70.82 ± 6.11	0.734
Forefoot width NLB (mm)	88.65 ± 6.46	88.68 ± 6.89	0.877	87.96 ± 5.37	87.52 ± 5.3	0.122
Hindfoot width NLB (mm)	63.87 ± 5.38	63.66 ± 5.48	0.242	62.27 ± 5.13	61.59 ± 5.06	0.009
Foot length LB (mm)	242 (233–255)	242 (234–255)	<0.001	245.42 ± 14.22	245.73 ± 13.52	0.365
First MTJ LB (mm)	180 (173–189)	181 (174–190)	0.011	180.53 ± 10.5	180.95 ± 9.98	0.218
Max ILA LB (mm)	53.64 ± 7.32	55.61 ± 7.12	<0.001	57.07 ± 8.31	58 ± 7.72	0.001
Midfoot width LB (mm)	77.07 (73.22–82.08)	78.25 (73.9–82.72)	<0.001	76.15 ± 6.61	76.04 ± 6.53	0.744
Forefoot width LB (mm)	91.64 ± 6.44	91.46 ± 6.75	0.436	90.92 ± 5.58	90.38 ± 5.55	0.027
Hindfoot width LB (mm)	66.52 (63.4–70.61)	67.06 (63.67–70.61)	0.866	66.01 ± 4.95	65.22 ± 5.24	0.002
Diff: Foot length (mm)	2 (0–4)	2 (1–4)	0.323	3 (2–5)	4 (2.25–4.75)	0.457
Diff: First MTJ (mm)	2 (0–4)	2 (0–4)	0.439	3 (2–5)	4 (2–6)	0.008
Diff: Max ILA (mm)	−3.85 (−5.87; −2.21)	−4.82 (−6.48; −2.86)	0.001	−4.61 (−6.8; −2.69)	−4.65 (−7.21; −2.97)	0.446
Diff. Midfoot width (mm)	4.31 (2.52–6.47)	4.52 (2.96–6.48)	0.558	4.72 (3.66–6.86)	5 (3.21–6.98)	0.828
Diff. Forefoot width (mm)	2.81 (1.63–4.23)	2.68 (1.17–3.96)	0.123	2.68 (1.84–4.03)	2.48 (1.39–4)	0.250
Diff. Hindfoot width (mm)	3.12(1.59–4.67)	3.21 (1.85–4.75)	0.080	3.74 ± 2.34	3.63 ± 2.08	0.704
Navicular drop NLB (mm)	51 (46–58)	50 (46–57)	0.010	53.3 ± 6.67	53.36 ± 6.68	0.879
Navicular drop LB (mm)	46 (40–52)	45 (40–50)	0.002	46.84 ± 6.63	46.38 ± 7.56	0.310
Diff: Navicular drop (mm)	5 (3–8)	6 (4–8)	0.317	6 (4–8)	6 (5–9.75)	0.061
FPI TOTAL (Score)	5 (1–8)	6 (1–8)	0.001	5 (3–7)	6 (3–9)	0.273

RA, rheumatoid arthritis; NLB, non-load-bearing; LB, load-bearing; MTJ, metatarsophalangeal joint; Max ILA, maximum height of the internal longitudinal arch; Diff, difference; FPI, Foot Posture Index, level of significance at *p* < 0.05.

**Table 2:** Symmetry comparison of patients with RA and those with foot pain and no RA using Symmetry Index.

	Non-RA Group (*n* = 235)		RA Group (*n* = 64)		
	Mean(%)	SD	Mean(%)	SD	*p* Value
Foot length NLB	0.05	0.97	0.40	1.92	0.15
Max ILA NLB	2.13	5.09	4.05	5.18	<0.01
Forefoot width NLB	−0.53	2.56	−0.07	3.87	0.37
Midfoot width NLB	−0.20	3.72	0.51	4.89	0.29

RA, rheumatoid arthritis; NLB, non-load-bearing; Max ILA, maximum height of the internal longitudinal arch; level of significance at *p* < 0.05.

**Table 3:** Comparison of the right and left feet in patients with RA ≤ 10 years vs. patients with RA > 10 years.

	RA ≤ 10 Years			RA > 10 Years		
	Right Foot	Left Foot		Right Foot	Left Foot	
	Mean/SD or Median/Interquartile Range *n* = 92		*p* Value	Mean/SD or Median/Interquartile Range *n* = 143		*p* Value
Foot length NLB (mm)	243 (233.25–256)	243 (235–256.75)	0.005	238 (229–248)	237 (230–248)	0.002
First MTJ NLB (mm)	180 (174.25–190)	181 (173–190)	0.163	178 (170–185)	177 (170–185)	0.121
Max ILA NLB (mm)	59.53 (54.59–64.63)	62.08 (57.41–66.58)	<0.001	56.05 (52.11–61.92)	58.8 (55.24–64)	<0.001
Midfoot width NLB (mm)	74.11 ± 6.13	74.23 ± 6.47	0.748	72.58 ± 6.53	73.29 ± 6.78	0.014
Forefoot width NLB (mm)	90.29 ± 5.86	90.17 ± 5.96	0.657	87.59 ± 6.63	87.72 ± 7.28	0.678
Hindfoot width NLB (mm)	64.64 ± 5.12	64.37 ± 5.47	0.344	63.38 ± 5.50	63.21 ± 5.45	0.459
Foot length LB (mm)	245 (236–260)	247 (236.25–260)	<0.001	240 (230–250)	240 (232–251)	0.005
First MTJ LB (mm)	183.5 (175–191.75)	185 (175–193.75)	0.011	179 (172–186)	180 (174–188)	0.216
Max ILA LB (mm)	55.39 ± 7.26	57.12 ± 6.91	<0.001	52.52 ± 7.16	54.63 ± 7.10	<0.001
Midfoot width LB (mm)	78.63 (73.43–82.77)	79.25 (74.78–83.45)	0.007	76.55 (73–81.02)	77.21 (73.27–82.09)	0.005
Forefoot width LB (mm)	93.45 ± 5.90	93.12 ± 5.57	0.274	90.47 ± 6.52	90.39 ± 7.23	0.805
Hindfoot width LB (mm)	67.23 (64.3–71.4)	68.14 (63.99–71.15)	0.871	66.11 (63.1–70.27)	66.66 (63.14–70.02)	0.830
Diff: Foot length (mm)	3 (0–4)	3 (2–4)	0.073	2 (0–4)	2 (0–4)	0.879
Diff: First MTJ (mm)	2 (0–4)	2.5 (0.25–4)	0.127	1 (0–4)	2 (0–3)	0.817
Diff: Max ILA (mm)	−3.62 (−6.39–−2.08)	−5.1 (−6.4–−3.24)	0.017	−3.91 (−5.74–−2.26)	−4.66 (−7.05–−2.56)	0.021
Diff. Midfoot width (mm)	4.21 (2.15–6.43)	4.8 (3.6–7.08)	0.096	4.32 (2.58–6.48)	4.49 (2.75–6.24)	0.538
Diff. Forefoot width (mm)	2.98 (1.72–4.75)	2.88 (1.41–4.48)	0.374	2.75 (1.53–3.93)	2.53 (1.14–3.52)	0.207
Diff. Hindfoot width (mm)	2.61 (1.72–4.13)	3.06 (1.96–4.26)	0.248	3.33 (1.57–5.06)	3.35 (1.73–5.03)	0.209
Navicular drop NLB (mm)	54 (49.25–60)	53 (48–60)	0.081	50 (45–56)	50 (45–55)	0.056
Navicular drop LB (mm)	50 (41–55)	47 (40.25–53)	0.006	45 (40–50)	45 (39–50)	0.073
Diff: Navicular drop (mm)	5 (2.25–8)	6 (4–8)	0.115	5 (4–8)	5 (3–8)	0.971
FPI TOTAL (Score)	4 (−1.75–7)	5 (1–8)	0.002	5 (2–8)	6 (2–9)	0.086

RA, rheumatoid arthritis; NLB, non-load-bearing; LB, load-bearing; MTJ, metatarsophalangeal joint; Max ILA, maximum height of the internal longitudinal arch; Diff, difference; FPI, Foot Posture Index, level of significance at *p* < 0.05.

**Table 4:** Symmetry comparison with Symmetry Index in patients with RA ≤ 10 years vs. patients with RA > 10 years.

	RA ≤ 10 Years(*n* = 92)		RA > 10 Years(*n* = 143)		
	Mean(%)	SD	Mean(%)	SD	*p* Value
Foot length NLB	0.38	1.55	0.41	2.14	0.99
Max ILA NLB	3.84	4.91	4.25	5.33	0.82
Forefoot width NLB	−0.19	2.98	0.01	4.36	0.91
Midfoot width NLB	−0.02	5.16	0.83	4.72	0.42

RA, rheumatoid arthritis; NLB, non-load-bearing; Max ILA, maximum height of the internal longitudinal arch; level of significance at *p* < 0.05.

## Data Availability

The data presented in this study are available on request from the corresponding author. The data are not publicly available to protect confidentiality of the research participants.
